# Case report: Circulating tumor DNA technology displays temporal and spatial heterogeneity in Waldenström macroglobulinemia during treatment with *BTK* inhibitors

**DOI:** 10.3389/pore.2023.1611070

**Published:** 2023-04-19

**Authors:** Jingjing Zhu, Xinyu Zhu, Fengyang Xie, Yi Ding, Huina Lu, Yan Dong, Ping Li, Jianfei Fu, Aibin Liang, Yu Zeng, Bing Xiu

**Affiliations:** ^1^ Department of Hematology, Tongji Hospital, Tongji University School of Medicine, Shanghai, China; ^2^ Department of Pathology, Tongji Hospital, Tongji University School of Medicine, Shanghai, China

**Keywords:** resistance, Waldenström macroglobulinemia, circulating tumor DNA (ctDNA), BTK inhibitor, ascites

## Abstract

**Background:** Waldenström macroglobulinemia (WM) is a rare subtype of B-cell lymphoma. Rituximab-based combination therapy and Bruton’s tyrosine kinase (*BTK*) inhibitors have greatly improved the prognosis of WM. Despite the high response rate and good tolerance of *BTK* inhibitors in treatment of WM, a proportion of patients still experience disease progression.

**Case presentation:** We report a 55-year-old man with relapsed WM. The patient achieved partial remission after six courses of CHOP chemotherapy and multiple plasma exchanges in initial treatment. He was admitted to the hospital with abdominal distension, and was diagnosed with relapsed WM and subsequently started on zanubrutinib. Disease progression and histological transformation occurred during treatment. We performed liquid biopsies on transformed plasma, tumor tissue and ascites at the same time and found high consistency between ascites and tissues. Moreover, we detected resistance mutations of *BTK* inhibitors (*BTK*, *PLCG2*) in ascites that were not detected in plasma or tissue. Eventually, the patient died during the 15-month follow-up after relapse.

**Conclusion:** We describe a rare case of WM transformation to DLCBCL treated with chemoimmunotherapy and *BTK* inhibition. We analyzed tumor DNA obtained at different anatomic sites and circulating tumor DNA (ctDNA) derived from plasma and ascites specimens, with apparent significant temporal and spatial heterogeneity. The case specifically highlights the clinical value of ctDNA of ascites supernatant from WM patients, which is a more convenient and relatively noninvasive method compared with traditional invasive tissue biopsy.

## Introduction

Waldenström macroglobulinemia (WM), a rare form of non-Hodgkin lymphoma, is a lymphoproliferative disease featuring bone marrow infiltration and IgM monoclonal gammopathy (1). WM represents 1%–2% of all hematological malignancies, and the morbidity is approximately five cases per million person-years. There are several clinical manifestations of this disease, including anemia, thrombocytopenia, hepatosplenomegaly, lymphadenopathy, and hyperviscosity (2). The somatic mutation *MYD88* ^*L265P*^ and mutations in *CXCR4* are mainly involved in the development of WM and play a crucial role in clinical features and overall survival (3). In general, updating of *MYD88* and *CXCR4* mutation profiling in WM has deepened the understanding of signaling pathways and promoted the development of *BTK* inhibitors. The 10th International Workshop for Waldenström’s macroglobulinemia (IWWM-10) recommended *BTK* inhibitors alone or in combination with rituximab as first-line treatment options for WM patients (4). With the advance of precision medicine, circulating tumor DNA (ctDNA) extracted from body fluids, including blood, cerebrospinal fluid, pleural effusion, and ascites, has been used for cancer genomic profiling (5–8). Resistance to *BTK* inhibitors secondary to histological transformation in WM is rare. Herein, we describe a patient with recurrent WM who developed histological transformation during treatment with zanubrutinib. We performed genetic testing of his pretransformed tissue and transformed ascites, plasma, and transformed tissue, and found a high degree of concordance between the ascites and transformed tumor tissue samples. This finding suggests that ascites may be used for liquid biopsy in WM to detect genetic mutations and early identification of clinical drug resistance.

## Case presentation

**FIGURE 1:**
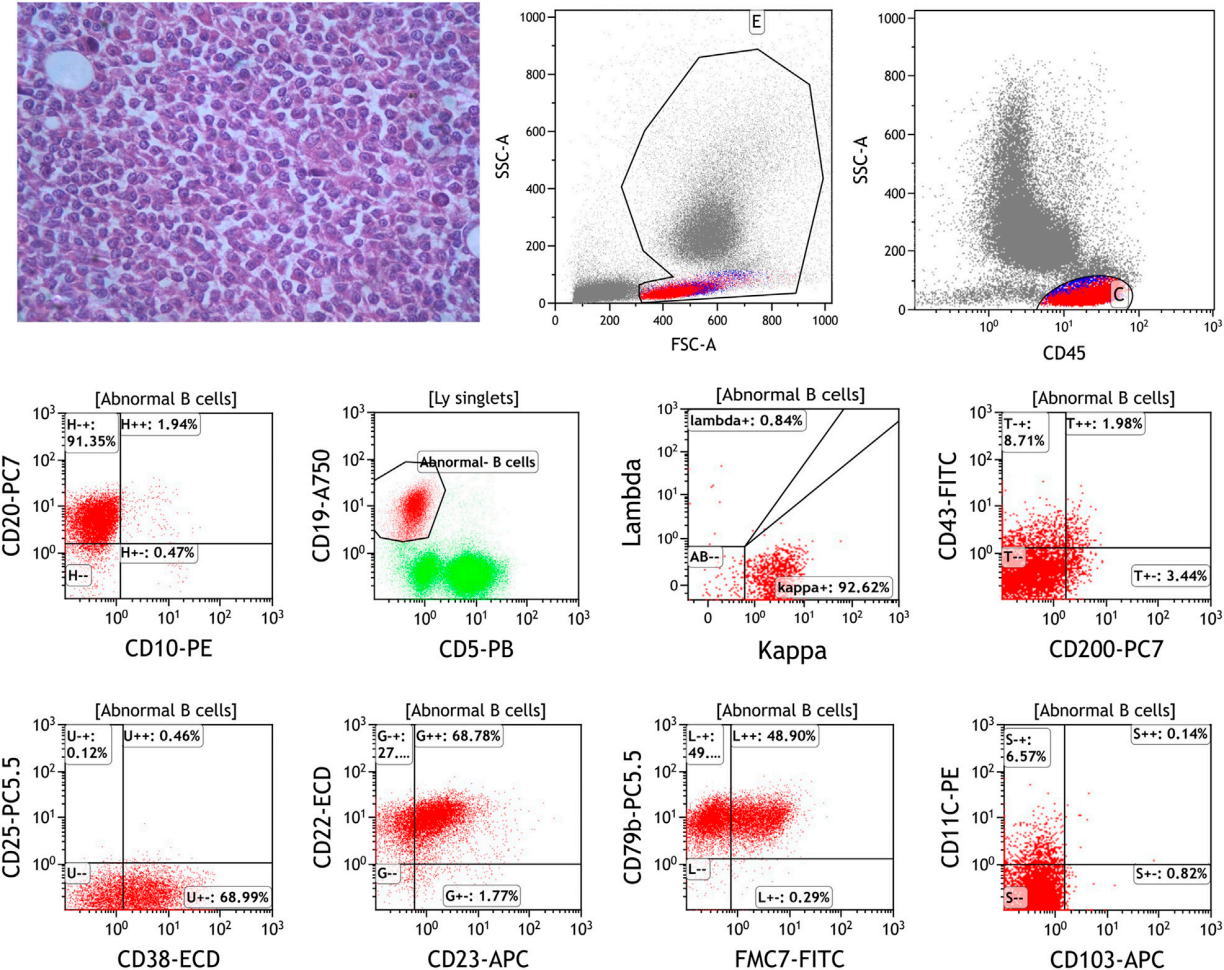
The bone marrow biopsy of the first disease recurrence showed the tumor cells composed of small lymphoid cells, plasmacytoid lymphocytes and plasma cells infiltrated the bone marrow (HEX400). Flow cytometric analysis of bone marrow specimen shows: CD20^+^, CD19^+^, CD5^−^, CD10^−^, Kappa^+^, Lambda^−^, CD200^−^, CD43^−^, CD38^+^, CD23p^+^, CD79B^+^, FMC7p^+^ and CD103^−^.

**FIGURE 2:**
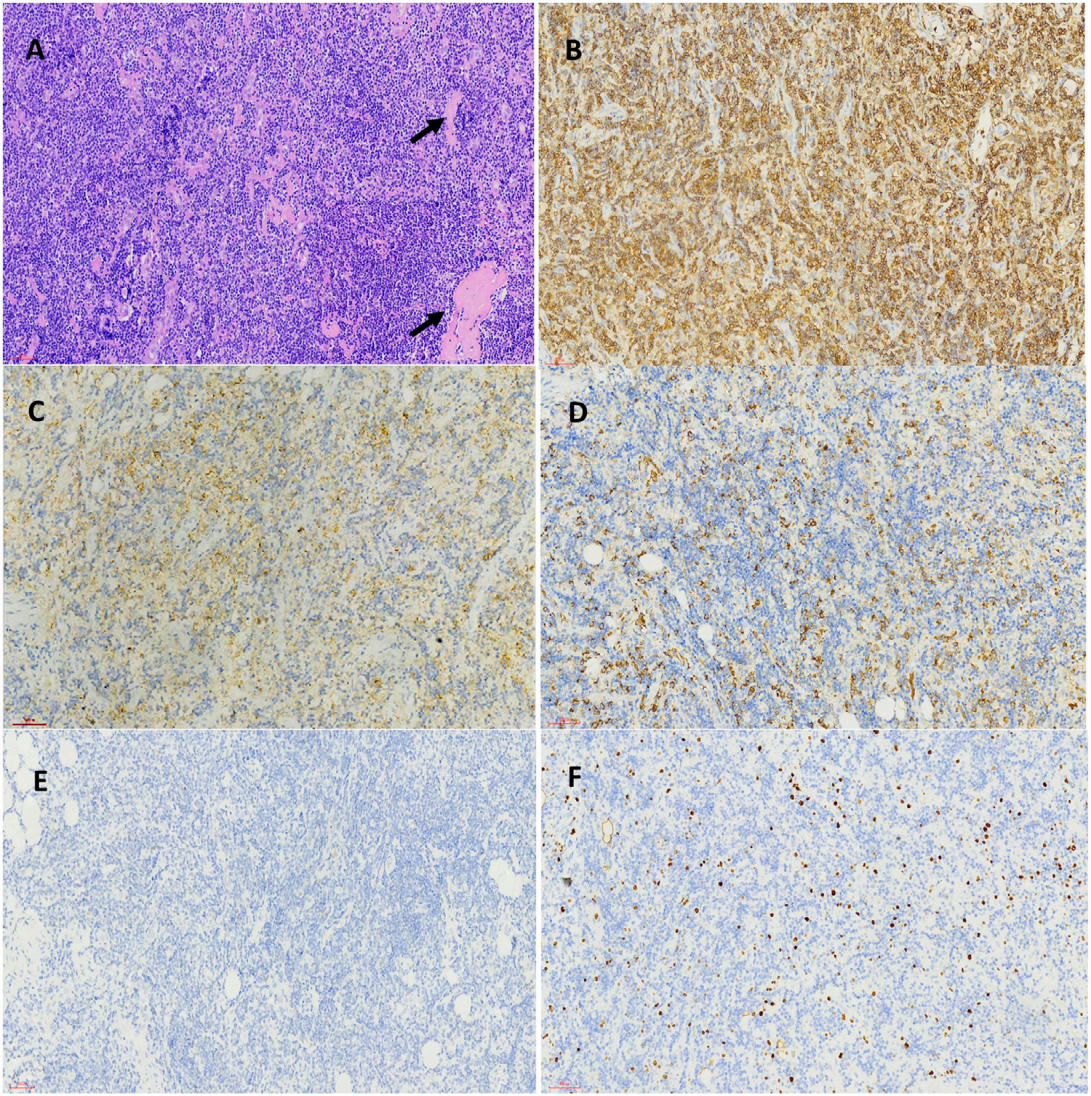
Pathological features of inguinal lymph node biopsy specimen before *BTK* inhibitor treatment. The structure of lymph node was destroyed and replaced by infiltration of small lymphocytes, amyloid material (black arrow) was present among tumor cells (HEX200) **(A)**. The inserted lower right picture was the magnification of tumor cells and amyloid material (HEX400). Immunohistochemical staining demonstrated the tumor cells were positive for CD20 **(B)**, CD38 **(C)**, IgM **(D)** and was negative for P53 **(E)** and showed a low proliferation rate by Ki-67 staining **(F)** (Envision X200).

**FIGURE 3:**
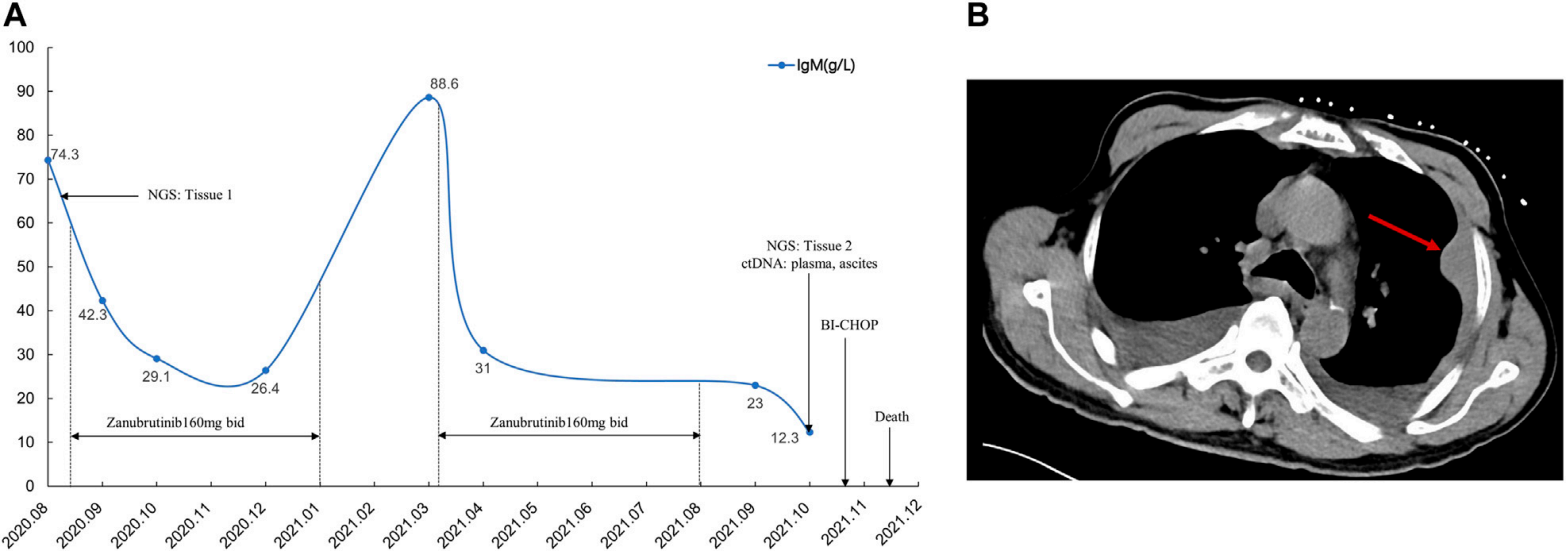
Treatment after disease recurrence and the time and type of obtained specimens over the disease course **(A)**. The red arrow shows chest wall mass on chest CT scan **(B)**.

A 55-year-old Asian man with a history of chronic hepatitis B visited the hospital in September 2013 due to recurrent dizziness, fatigue, pyrexia and night sweats for approximately 2 months. Clinical examination showed superficial lymphadenopathy and splenomegaly, with no tenderness. Laboratory examination results were as follows: hemoglobin, 40 g/L (reference range, 110–160 g/L); platelets, 130×10^9^/L (reference range, 100–300 × 10^9^/L); serum β-2 microglobulin, 3.85 mg/L (reference range, ≤2.80 mg/L); IgM at 67.7 g/L; (reference range, 0.4–3.0 g/L); Coombs’ test (+). An IgM-kappa monoclonal peak was observed by serum immunofixation electrophoresis. Bone marrow biopsy showed diffuse trabecular space invasion by small lymphocytes differentiated by plasma cells and confirmed the diagnosis of WM. The international prognostic scoring system for the Waldenström macroglobulinemia (IPSS WM) score was 2, indicating intermediate risk. In view of the anemia, extremely elevated IgM and hyperviscosity present, the patient received six courses of CHOP chemotherapy (R-CHOP was used once but was discontinued due to allergy) and multiple plasma exchanges. He achieved partial remission after treatment with the level of IgM falling to 30.2 g/L from November 2013 to May 2017. However, the patient did not regularly attend follow-up in the next year, even though he had recurrent abdominal discomfort. He developed increasing abdominal distention and was admitted to our department in August 2020. Physical examination revealed emaciation, anemic appearance, multiple enlarged lymph nodes in the cervical, axillary and inguinal areas, frog belly, splenomegaly, shifting dullness (+), fluid thrill and edema of the bilateral lower limbs. Laboratory examinations showed WBC at 2.83 × 10^9^/L, hemoglobin at 85 g/L, platelets at 94 × 10^9^/L, serum β-2 microglobulin at 6.68 mg/L, and IgM at 74.32 g/L. An IgM-kappa monoclonal peak was still observed by serum immunofixation electrophoresis. Bone marrow biopsy showed that tumor cells composed of small lymphoid cells, plasmacytoid lymphocytes and plasma cells had infiltrated the bone marrow. The bone marrow flow cytometry results were as follows: CD19^+^, CD20^+^, CD79B+, CD38^+^, CD138−, CD5−, CD10−, CD103−, Kappa+, Lambda−, CD200^−^, CD43^−^, FMC7p+, and CD23p+ (Figure 1). Immunohistochemical (IHC) analysis of the left inguinal lymph node biopsy showed that the tumor cells were positive for CD20, CD19, CD38, CD138, and IgM and negative for CD5, CD10, kappa, lambda and p53, which was consistent with the diagnosis of WM, with Ki-67 = 15% (Figure 2). Given the recurrence of the disease, the patient was treated with 160 mg zanubrutinib twice daily, continuous intraperitoneal drainage and albumin infusion. During the 4 months after administration of zanubrutinib, the patient experienced three infections (*Escherichia coli* septicemia once and left femoral periostitis two times). His hemoglobin level returned to 126 g/L, and his IgM level dropped to 26.4 g/L, with shrinkage of the spleen and retroperitoneal lymph nodes after treatment, thus achieving partial remission in December 2020 (Figure 3).

**FIGURE 4:**
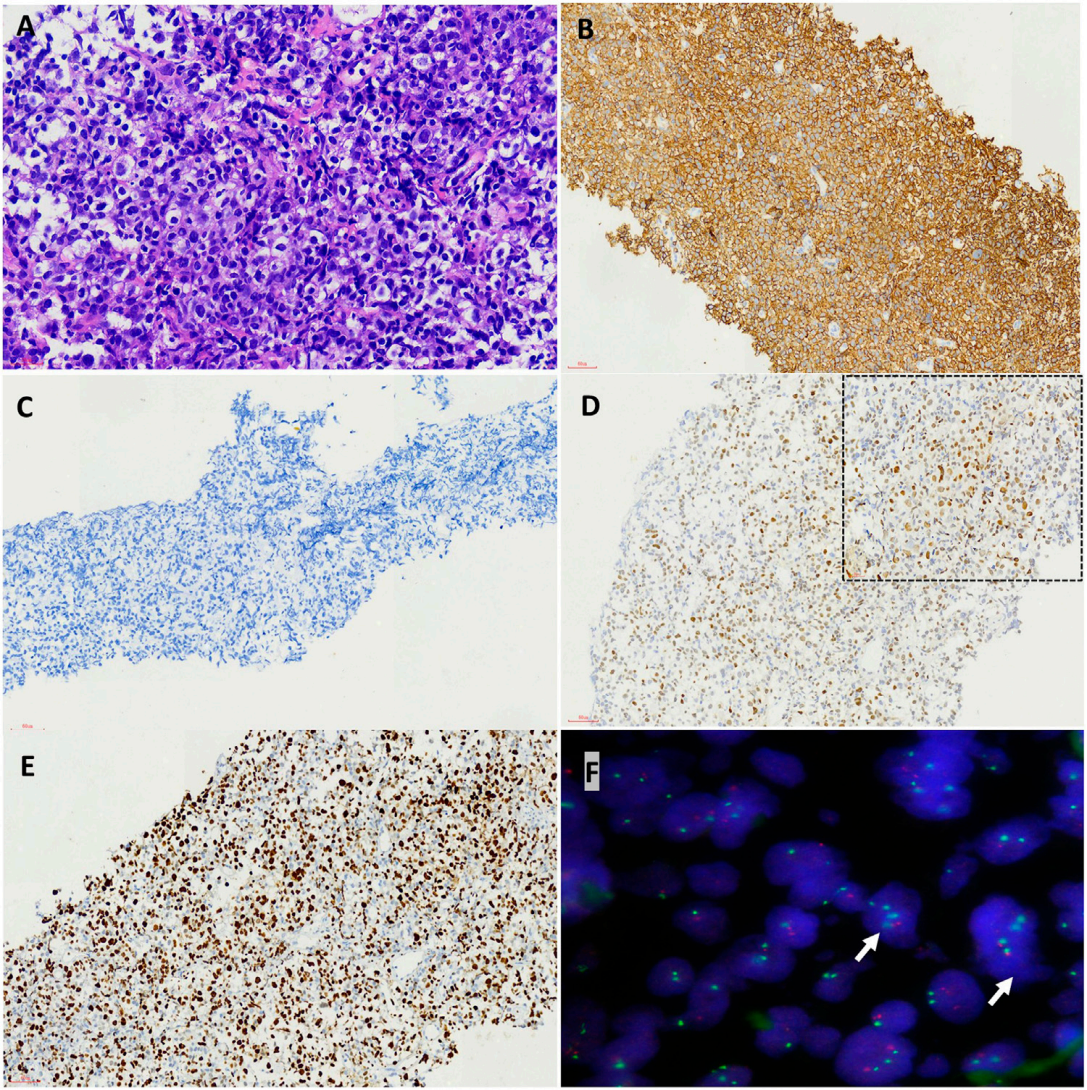
Pathological features of high grade transfomation in the biopsy from the chest wall after *BTK* inhibitor treatment. The tumor cells were medium to large in size with round or oval nuclei and small nucleoli could be seen in some tumor cells (HEX400) **(A)**. Immunohistochemical staining demonstrated the tumor cells was positive for CD20 **(B)** but negative for CD38 **(C)**, diffusely and strongly positive for P53 (D, inserted upper right picture was high power view, X400) and showed a much higher proliferation rate by Ki-67 staining **(E)** (Envision X200). FISH test showed P53 gene lost (white arrow, the red signal was P53 gene, the green signal was CEP17) **(F)**.

**FIGURE 5:**
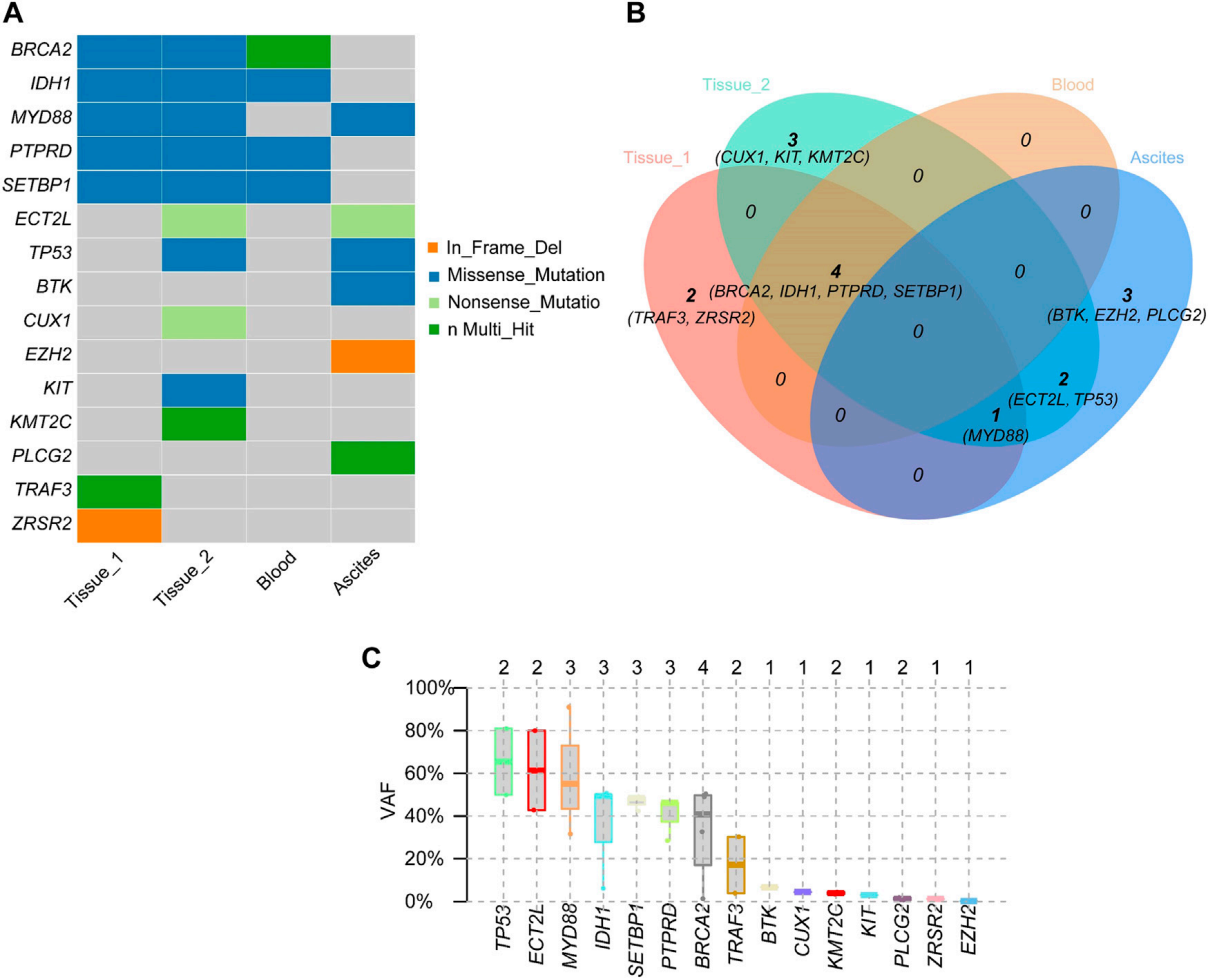
Mutated genes and gene mutation types of inguinal lymph node (Tissue 1), transformed chest wall tissue (Tissue 2), peripheral blood, and ascites **(A)**. Relationships of mutant genes in different specimens **(B)**. Variant Allele Frequency of mutated genes **(C)**.

Two months after zanubrutinib self-withdrawal, the patient was hospitalized with fever and pain in the left lower limb in March 2021. Laboratory examinations revealed hemoglobin at 105 g/L, platelets at 115 × 10^9^/L, serum β-2 microglobulin at 8.32 mg/L and IgM at 88.6 g/L. HBV DNA quantification <1.0^e+2^ IU/mL. Imaging examination confirmed enlargement of the spleen and retroperitoneal lymph nodes. Considering that the patient’s IgM increased significantly after drug withdrawal and disease progression, we restarted 160 mg zanubrutinib twice daily. However, at 1.5 months after zanubrutinib self-withdrawal, he developed worsening abdominal distention and was readmitted in September 2021. Laboratory examinations revealed hemoglobin at 133 g/L, platelets at 93*10^9^/L, IgM at 23.0 g/L, serum β-2 microglobulin at 5.11 mg/L; HBV DNA quantification <5.99^e+6^ IU/mL, and serum-ascites albumin gradient (SAAG) at 19.7 g/L. Gastroscopy and imaging examination confirmed chronic liver cirrhosis. Notably, chest CT showed a new mass in the left chest wall (8.5 × 3.1 cm) (Figure 3). Chest wall tissue biopsy showed that the tumor cells were medium to large in size with round or oval nuclei, and small nucleoli could be seen in some tumor cells. IHC demonstrated that the abnormal lymphoid cells were CD19^+^, CD20^+^, CD10^−^, Bcl-6^+^, MUM^+^, Bcl-2^+^, CD38^−^, CD138^−^, CD5^−^, and CD23^−^. The tumor cells were diffusely and strongly positive for P53. A high Ki-67 index (>90%) indicated aggressive lymphoma. Fluorescence *in situ* hybridization test showed that the P53 gene was lost (Figure 4). According to Hans’ criteria, these findings suggest that the patient had a non-GCB subtype of DLBCL. The international prognostic index was 4, indicating high risk. From the clinical perspective, we considered that the WM had transformed to DBCL. However, clonal homology validation was not performed because of insufficient samples and disease progression. We analyzed tumor DNA obtained from different anatomic sites (left inguinal lymph node, chest wall tissue) and ctDNA derived from plasma and ascites supernatant, which showed significant temporal and spatial heterogeneity. High-throughput sequencing analysis was performed using the whole exome region of 193 cancer-related genes (ChosenMed, Beijing, China) with MGISEQ-2000, with a mean sequencing depth of 500× and 10000× in tissue and ctDNA, respectively (Supplementary Table S1). Data were analyzed using an in-house bioinformatics pipeline. According to the NGS results, *MYD88* ^*L265P*^ was detected in left inguinal lymph node; *MYD88* ^*L265P*^, *TP53* ^*Y220D*^ and *ECT2L* ^R255*^were detected in the chest wall tissue. In addition to these mutations, we detected genetic mutations (*BTK* ^*T316A*^, *PLCG2* ^*D334G,R337W*^) associated with resistance to *BTK* inhibitors in ctDNA derived from ascites (Figure 5). However, these mutations were not detected in plasma ctDNA. Genetic testing revealed high concordance between ascites and tumor tissue, suggesting the clinical utility of ascites for liquid biopsy in WM patients. Considering his performance status and financial situation, the patient received bendamustine, ibrutinib 140 mg twice daily (reduced due to liver damage), attenuated CHOP (cyclophosphamide, pharmorubicin, vinorelbine, and dexamethasone) and antiviral therapy (entecavir). However, he did not respond to chemotherapy, and there was no significant improvement. Finally, he succumbed to progressive disease and poor performance status in November 2021. The overall survival time was 98 months.

## Discussion

WM is a B-cell malignancy manifested by accumulation of lymphoplasmacytic cells in bone marrow and/or lymphatic tissue. Elevated serum immunoglobulin-M (IgM) tends to correlate with disease burden and can cause symptoms associated with hyperviscosity, tissue infiltration, and autoimmune phenomena (9). WM has been associated with a variety of highly recurrent somatic mutations, including in *MYD88* (95%–97%), *CXCR4* (30%–40%), *ARID1A* (17%), and *CD79B* (8%–15%). These identifications of somatic mutations provide important insight into diagnosis, drug options for treatment, and monitoring of disease progression in WM (10). In recent years, precision medicine has gained popularity, integrating genetic testing into clinical care to maximize patient outcomes. The use of appropriate samples for specific genetic analysis is particularly important for patients who cannot undergo invasive procedures. Compared with traditional tissue biopsy, circulating tumor DNA (ctDNA) (commonly known as liquid biopsy) is a relatively noninvasive method that can be used for diagnosis, dynamic monitoring of therapeutic effects, revealing the emergence of drug resistance and quantification of minimal residual disease (MRD) in cancer. Numerous body fluids can be used for detection of ctDNA, including blood, urine, saliva, cerebrospinal fluid, pleural fluid and bronchial washings (11). Liquid biopsy is increasingly utilized in treatment of hematologic malignancies and has made great contributions. Many studies have illustrated the potential of liquid biopsy approaches for diagnosis, therapy, assessment of MRD, early detection of relapse and prognosis in aggressive B-cell lymphomas, acute myeloid leukemia, myelodysplastic syndrome, chronic lymphocytic leukemia and multiple myeloma (12–15). This cost-efficient method can prevent the discomfort and complexity caused by repeated bone marrow biopsy in WM. Some recent studies have shown that it is feasible to assess genomic landscapes with this minimally invasive, yet sensitive tool (5, 16). Bagratuni *et al.* showed higher MAFs of *MYD88* ^*L265P*^ in both peripheral blood ctDNA and bone marrow tDNA in symptomatic WM, with MAF being reduced after therapy and increased in relapsed by Cast-PCR (17). In addition to peripheral blood, *MYD88* ^*L265P*^mutation has been detected in skin lesions, pleural effusions and CSF in WM (18–20), thought it is rarely reported in ascites. Compared to tumor tissue and plasma, the DNA detected in ascites supernatants may derive from necrotic or apoptotic tumor cells and contain abundant tumor-derived DNA, which provides more important genomic information for different tumors (8, 21, 22). In our study, the gene mutations detected in ctDNA from ascites supernatants and chest wall tumor tissue were highly identical, whereas no significant genetic mutations were detected in plasma ctDNA. These results indicate that ascites supernatants may be suitable sample types in clinical practice for genetic and molecular analyses for WM patients with ascites.

Unexpectedly, we also detected genetic mutations (*BTK* ^*T316A*^and *PLCG2* ^*D334G, R337W*^) associated with *BTK* inhibitor resistance in ascites derived ctDNA. *BTK* inhibitors are new treatment options for WM patients. Ibrutinib was the first *BTK* inhibitor to receive approval by the FDA for treatment of WM (23). Although ibrutinib has high therapeutic activity and leads to durable remission in WM treatment, its use has been affected by off-target effects and drug resistance (24). Compared with ibrutinib, zanubrutinib is a selective, irreversible *BTK* inhitior, that has less toxicity and better response quality (25). With the increasing use of *BTK* inhibitors, more WM patients experience resistance to treatment. Acquired resistance to ibrutinib is strongly associated with recurrent mutations in *BTK* (especially *BTK* ^*Cys481*^) and *PLCG2*. *BTK* ^*T316*^, a mutation in the SH2 nonkinase domains of *BTK*, has been reported in CLL patients who experienced progression on ibrutinib. This mutation prevents critical contact with phosphotyrosine, thereby reducing the affinity of *BTK* to BLNK or other *BTK* partner proteins and leading to drug resistance. Sharma et al. identified that cells carrying *BTK* ^*T316A*^ exhibit resistance to ibrutinib at the cellular and molecular levels similar to those carrying *BTK* ^*C481S*^ (26). Studies in CLL and WM have shown that gain-of-function mutations in *PLCG2* ^*(R665W, L845F, S707)*^ are associated with ibrutinib resistance (27–29). Chen et al. confirmed that the *BTK* ^*C481S*^ mutation confers resistance to ibrutinib by activating ERK1/2 continuously and releasing inflammatory and prosurvival cytokines in *MYD88-*mutated WM; (30). Recently, *PLCG2* ^*D334G*^ was detected in the TIM domain of ibrutinib-resistant R/R CLL (31) and may be linked to Richter’s transformation (32). However, *PLCG2* ^*(D334G, R337W)*^ mutations have not been reported in WM. In our case, the reason why the patient developed drug resistance and histological transformation may be related to treatment-driven clonal evolution. D. Talaulikar et al. suggested that *IGHV* analysis is required to determine whether DLBCL tissue is clonally associated with the previous WM clone. It is important to determine whether the two tumors share the same clonal origin, as patients with clonal homologous histological transformations tend to have worse prognosis (33). Unfortunately, *IGHV* analysis of the patient was limited by the paucity of samples. Whether chest wall tumors have clonal homology or tend to be of independent clonal origin remains speculative. We detected the mutation of *TP53* in chest wall tumor and its VAF was 80.98%. *TP53* mutation predicts unfavorable prognosis despite the low frequency in WM. Poulain *et al.* observed that 7% of WM patients had *TP53* mutation at diagnosis and more genomic abnormalities. Of note, the overall survival of WM with *TP53* alterations was significantly short, especially in those with symptomatic WM, which was independent of the IPSSWM score (34). Disease progression, complications and development to high-grade lymphoma are the main causes of death in WM (35). Histological transformation of WM to DLBCL may occur rarely. Lin et al. reported that histological transformation to DLBCL occurs in 13% of WM patients, which is related to poor clinical prognosis (36). A large retrospective study of 1,466 WM patients described that WM has an approximately 2% cumulative occurrence rate of histological transformation after 10 years, with a median survival of 2.7 years (37). Durot et al. suggested that the presence of elevated LDH, constitutional symptoms and extranodal involvement, especially MYD88-associated sites, should be highly suspected of histological transformation in WM patients (38). Moreover, Jiménez et al revealed that disease transformation is largely determined by the frequency and specificity of acquired alterations. Some acquired recurrent mutations (such as in *PIM1*, *FRYL* and *HNF1B*) may be involved in clonal selection for disease transformation. There is potential to predict transformation risk with *CD79B* in WM (39). In our case, transformed DLBCL was highly aggressive and was associated with adverse prognostic factors, a high IPI score, extranodal involvement and a high Ki-67 index at diagnosis. The patient had extremely poor prognosis and died within 1.5 months after histological transformation. The course of dynamic change in IgM and comparison of pathological characteristics by both indolent and aggressive processes, and the presence of multiple genetic mutation sites supports disease progression and histological transformation to DLBCL.

## Conclusion

In conclusion, ctDNA derived from ascites provides more information for genetic profiling and drug resistance than plasma or tissue. In the absence of tumor tissues, ascites may be a good alternative for genetic analysis in clinical practice in WM patients with ascites. We believe that ctDNA analysis should be performed in prospective studies to assess genomic profiling of disease processes in WM. Although clonal homology between DLBCL and antecedent WM is not clear, analysis of genetic mutations and aggressive histologic transformation features strongly suggests a clonal relationship between the two B-cell lymphomas. Since the prognosis of transformed DLBCL is worse than that of *de novo* DLBCL, it is important to evaluate the clonal relationship between antecedent WM and transformed DLBCL.

## Data Availability

The original contributions presented in the study are included in the article/Supplementary Material, further inquiries can be directed to the corresponding authors.
