# Supplementary material for: Case report: Circulating tumor DNA technology displays temporal and spatial heterogeneity in Waldenström macroglobulinemia during treatment with BTK inhibitors
Source: Pathol Oncol Res. 2023 Apr 19;29:1611070. doi: 10.3389/pore.2023.1611070 (PMC10154527; doi:10.3389/pore.2023.1611070)
Supplement: Supplementary file 1 [file Table1.DOCX]

**Chest wall tissue**

| Mutated gene | Transcript ID | Mutated site | Nucletic change | Amino acid change | dbSNP | VAF(%) | Depth |
| --- | --- | --- | --- | --- | --- | --- | --- |
| The pathogenic and likely pathogenic mutation | | | | | | | |
| CUX1 | NM_181552 | exon5 | c.292G>T | p.G98* | - | 4.40 | 341 |
| ECT2L | NM_001077706 | exon7 | c.763C>T | p.R255* | rs753798507 | 80.00 | 125 |
| MYD88 | NM_002468 | exon5 | c.794T>C | p.L265P | rs387907272 | 91.02 | 256 |
| TP53 | NM_000546 | exon6 | c.658T>G | p.Y220D | rs530941076 | 80.98 | 163 |
| Variants of uncertain clinical significance | | | | | | | |
| BRCA2 | NM_000059 | exon7 | c.572A>T | p.D191V | rs397507798 | 32.68 | 257 |
| IDH1 | NM_001282386 | exon3 | c.59G>A | p.R20Q | rs201258988 | 6.14 | 342 |
| KIT | NM_000222 | exon14 | c.2009C>A | p.T670K | - | 3.08 | 389 |
| KMT2C | NM_170606 | exon2 | c.221C>A | p.T74K | - | 3.47 | 375 |
| KMT2C | NM_170606 | exon2 | c.236C>A | p.T79K | - | 4.42 | 294 |
| PTPRD | NM_002839 | exon21 | c.1202G>A | p.R401Q | - | 28.51 | 221 |
| SETBP1 | NM_015559 | exon4 | :c.2383A>C | p.T795P | - | 42.32 | 371 |

**Left inguinal lymph node**

| Mutated gene | Transcript ID | Mutated site | Nucletic change | Amino acid change | dbSNP | VAF(%) | Depth |
| --- | --- | --- | --- | --- | --- | --- | --- |
| The pathogenic and likely pathogenic mutation | | | | | | | |
| MYD88 | NM_002468 | exon5 | c.794T>C | p.L265P | rs387907272 | 31.60 | 1864 |
| TRAF3 | NM_003300 | exon3 | c.263_272del | p.C88fs | - | 30.23 | 1148 |
| Variants of uncertain clinical significance | | | | | | | |
| BRCA2 | NM_000059 | exon7 | c.572A>T | p.D191V | rs397507798 | 49.04 | 1413 |
| IDH1 | NM_001282386 | exon3 | c.59G>A | p.R20Q | rs201258988 | 50.68 | 1618 |
| TRAF3 | NM_003300 | exon4 | c.343A>T | p.I115F | - | 3.92 | 1608 |
| ZRSR2 | NM_005089 | exon11 | c.1338_1343del | p.S447_R448del | rs768273733 | 1.24 | 1289 |
| PTPRD | NM_002839 | exon21 | c.1202G>A | p.R401Q | - | 46.25 | 1228 |
| SETBP1 | NM_015559 | exon4 | :c.2383A>C | p.T795P | - | 48.33 | 1291 |

**the ctDNA of ascites** **supernatant**

| Mutated gene | Transcript ID | Mutated site | Nucletic change | Amino acid change | dbSNP | VAF(%) | Depth |
| --- | --- | --- | --- | --- | --- | --- | --- |
| The pathogenic and likely pathogenic mutation | | | | | | | |
| BTK | NM_000061 | exon11 | c.946A>G | p.T316A | - | 6.71 | 1297 |
| ECT2L | NM_001077706 | exon7 | c.763C>T | p.R255* | rs753798507 | 42.80 | 1278 |
| MYD88 | NM_002468 | exon5 | c.794T>C | p.L265P | rs387907272 | 55.06 | 1938 |
| PLCG2 | NM_002661 | exon12 | c.1001A>G | p.D334G | - | 1.78 | 1687 |
| PLCG2 | NM_002661 | exon12 | c.1009C>T | p.R337W | - | 0.79 | 1775 |
| TP53 | NM_000546 | exon6 | c.658T>G | p.Y220D | rs530941076 | 49.92 | 1308 |
| Variants of uncertain clinical significance | | | | | | | |
| EZH2 | NM_004456 | exon6 | c.566_568del | p.D189del | rs587778303 | 0.21 | 2408 |

**the ctDNA of plasma (Peripheral Blood)**

| Mutated gene | Transcript ID | Mutated site | Nucletic change | Amino acid change | dbSNP | VAF(%) | Depth |
| --- | --- | --- | --- | --- | --- | --- | --- |
| Variants of uncertain clinical significance | | | | | | | |
| BRCA2 | NM_000059 | exon7 | c.572A>T | p.D191V | rs397507798 | 50.40 | 1605 |
| IDH1 | NM_001282386 | exon3 | c.59G>A | p.R20Q | rs201258988 | 49.41 | 1429 |
| PTPRD | NM_002839 | exon21 | c.1202G>A | p.R401Q | - | 46.16 | 2901 |
| SETBP1 | NM_015559 | exon4 | c.2383A>C | p.T795P | - | 49.07 | 2945 |
